# Supplementary material for: Impact of Integration of Severe Acute Malnutrition Treatment in Primary Health Care Provided by Community Health Workers in Rural Niger
Source: Nutrients. 2021 Nov 14;13(11):4067. doi: 10.3390/nu13114067 (PMC8625976; doi:10.3390/nu13114067)
Supplement: Supplementary file 1 [file nutrients-13-04067-s001.zip › nutrients-1409947-supplementary.pdf]

Figure S1. Sanitary map Mayahi district.

Niger map. Maradi Region. District Mayahi

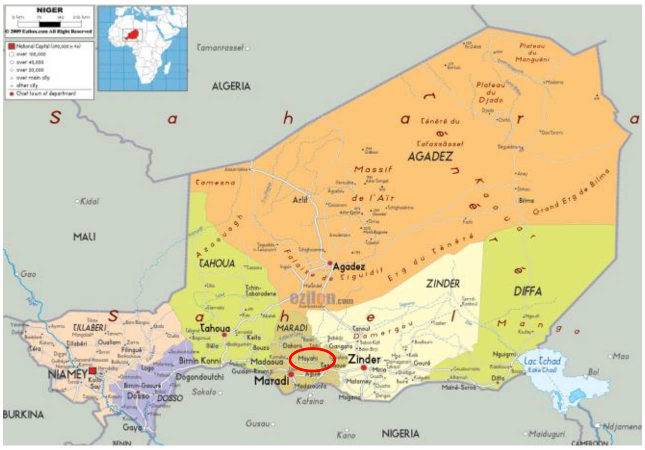

Sanitary map Mayahi district.

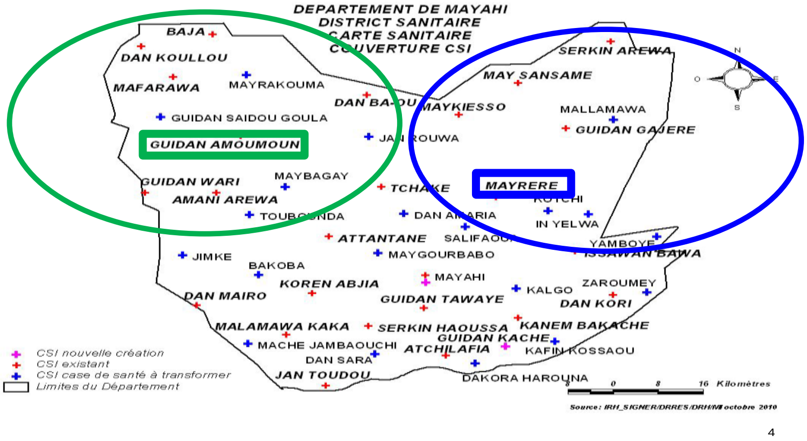

Control zone, Mayrere : Total population 64,183 hab. Children under five 12,836

4 Health facilities: Meireyey, Guidan gagere, Serkin arewa, Messanssame

Intervention zone, Guidan Amoumoun : Total population 88,199 hab. Children under five 17,639

6 Health facilities: Guidan amoumoune, Guidan wari, Baja, Mafarawa, Dan koullo, Dan mallan merakouma

10 Community Health Workers: Guidan barmo, Na aya, Koudou Baraou, Guidan losso, Dadin tamro, Jibbi saboua, Kouka dan bako, Dan ladi bakane, Melawa, Malloumey
